# Risk of Foot-and-Mouth Disease Spread Due to Sole Occupancy Authorities and Linked Cattle Holdings

**DOI:** 10.1371/journal.pone.0035089

**Published:** 2012-04-19

**Authors:** Richard J. Orton, Paul R. Bessell, Colin P. D. Birch, Anthony O'Hare, Rowland R. Kao

**Affiliations:** 1 Boyd Orr Centre for Population and Ecosystem Health, Institute of Biodiversity, Animal Health and Comparative Medicine, College of Medical, Veterinary and Life Sciences, University of Glasgow, Glasgow, United Kingdom; 2 Veterinary Laboratories Agency, New Haw, Addlestone, Surrey, United Kingdom; Massey University, New Zealand

## Abstract

Livestock movements in Great Britain are well recorded, have been extensively analysed with respect to their role in disease spread, and have been used in real time to advise governments on the control of infectious diseases. Typically, livestock holdings are treated as distinct entities that must observe movement standstills upon receipt of livestock, and must report livestock movements. However, there are currently two dispensations that can exempt holdings from either observing standstills or reporting movements, namely the Sole Occupancy Authority (SOA) and Cattle Tracing System (CTS) Links, respectively. In this report we have used a combination of data analyses and computational modelling to investigate the usage and potential impact of such linked holdings on the size of a Foot-and-Mouth Disease (FMD) epidemic. Our analyses show that although SOAs are abundant, their dynamics appear relatively stagnant. The number of CTS Links is also abundant, and increasing rapidly. Although most linked holdings are only involved in a single CTS Link, some holdings are involved in numerous links that can be amalgamated to form “CTS Chains” which can be both large and geographically dispersed. Our model predicts that under a worst case scenario of “one infected – all infected”, SOAs do pose a risk of increasing the size (in terms of number of infected holdings) of a FMD epidemic, but this increase is mainly due to intra-SOA infection spread events. Furthermore, although SOAs do increase the geographic spread of an epidemic, this increase is predominantly local. Whereas, CTS Chains pose a risk of increasing both the size and the geographical spread of the disease substantially, under a worse case scenario. Our results highlight the need for further investigations into whether CTS Chains are transmission chains, and also investigations into intra-SOA movements and livestock distributions due to the lack of current data.

## Introduction

Explicit network data are increasingly being used for the development of mathematical models to inform disease control [1], [2], [3], [4], [5]. In Great Britain (GB), livestock movements are well recorded and describe the dynamic network of connections among livestock holding locations. These connections can be critical for disease spread, as was shown in the 2001 epidemic of foot-and-mouth disease (FMD) in the United Kingdom (UK) [6]. Control of the 2001 FMD epidemic resulted in approximately 8.5 million livestock culled, and is estimated to have cost GBP 4–6 billion [7]. Since 2001, a number of policies have been introduced to reduce the possible impact of the reintroduction of FMD, including movement standstills for holdings in receipt of animal movements [8].

The Cattle Tracing System (CTS) records the movements of cattle at the individual level for all of GB, whilst the Animal Movements License System (AMLS) records the movements of other large livestock (e.g. sheep, pigs) at the batch level in England and Wales; in Scotland, a database equivalent to AMLS is held by the Scottish Animal Movements Unit (SAMU). Movement data have been extensively utilised for modelling the spread of infectious diseases of livestock [1], [2], [3], [4], [5]. However, movements represent just one mechanism by which infectious diseases can spread [3], [6], [9], and the utility of additional detailed data is at least partially dependent on the importance of alternative mechanisms of transmission.

Livestock holding locations in GB are assigned a unique County Parish Holding (CPH) number. Typically such holdings are treated as distinct entities that must observe movement standstills upon receipt of any livestock (6 days for cattle and sheep in both England/Wales, 13 days in Scotland, and 20 days for pigs throughout GB [8]), and must report any livestock movements to CTS or AMLS. However, there are currently two dispensations that can exempt holdings from either observing standstills or reporting movements.

A group of holdings within the same management and control may be granted a Sole Occupancy Authority (SOA, [10]). When sheep, pigs and cattle are brought onto any one of the holdings within the SOA a standstill is imposed on all the holdings in the SOA. However, animals may move between holdings inside the SOA without observing the standstill, allowing infection to spread rapidly amongst SOA members. Although there is a requirement for all intra-SOA movements to be reported, the 5 mile rule for sheep and goats previously exempted the reporting of movements between land that is within 5 miles and under the same control; we note that the 5 mile rule for sheep and goats has recently been disbanded in England [11] and now all movements between different CPHs must be reported. However, the reporting of intra-SOA cattle movements to CTS has always been required. A holding can only be a part of one SOA at a time, restricting the extent to which SOAs can create bridges across the community; however, there are currently no distance limits between holdings in the SOA. Thus SOAs may not only increase the risk of disease spread between the holdings, but may also link otherwise unconnected ‘communities’ within the livestock movement network, as such communities typically show strong spatial aggregation [12].

Cattle keepers may apply to the British Cattle Movement Service (BCMS) to have two CPH numbers linked on CTS for the purpose of exempting them from reporting movements of cattle between the two linked CPHs, thus creating what is called a “CTS Link” [10]. There is currently no limit to the number of CTS Links that an individual holding can be in. However, farmers must still record movements between linked holdings in their herd farm records. There are two different types of CTS Links [10]: (1) Shared Facilities (SF's) – links between holdings under the same ownership which share facilities, these links must be permanent (longer than 364 days), the movements frequent, and holdings must be within 25 miles of each other; and (2) Additional Land (AL's) – links between holdings for summer grazing and winter housing, there is no mileage restriction, but the link is temporary and must be renewed after 364 days. The existence of CTS Links can increase: (1) The risk of disease spread because of increased contiguity and because standstill violations are difficult to police; (2) The difficulty of tracing any cattle because movements over potentially long distances are not reported to CTS; and (3) The number of holdings which may be assumed to be contiguous to an infected holding (linked farms would be assumed contiguous to one another), thereby increasing the costs associated with containing a disease outbreak.

In order to assess the additional risk associated with these exemptions, we investigate if usages of SOAs and CTS Links are substantial and increasing, we identify other characteristics of holdings involved in such linkages, and then use computational modelling to assess the potential impact of such linked holdings on both the overall size and geographical spread of an FMD epidemic. In our previous modelling work, the inclusion of SOAs had only a minimal impact on final epidemic size {Green,2006}. However, the increased number of SOAs since then and the additional effect of CTS Links merits further investigation of their impact on FMD spread.

## Results

### Data Analysis - SOAs

**Figure 1:**
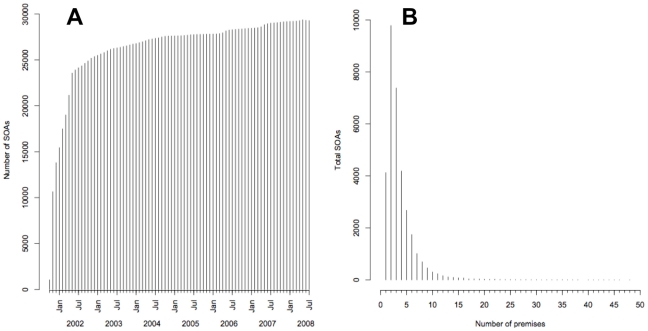
Number of SOAs in existence over time and the distribution of component holdings. (A) Shows the monthly number of SOAs in existence from their inception in late 2001 to mid-2008. (B) Shows the distribution of the number of component holdings that comprise each SOA; the x-axis has been truncated to remove the outlier SOA containing 250 holdings for display purposes.

Data on all SOAs in England and Wales were combined with census data and AMLS animal movements for an analysis of SOAs and the holdings that comprise them. As can be seen in Figure 1A, the number of SOAs in England/Wales has been steadily increasing over time. There was an initial rapid increase in the number of SOAs during the first 9 months they were in existence to just under 25,000 SOAs, with subsequent steady increases to around 29,000 at the start of 2008, consisting of just over 100,000 distinct holdings. SOAs typically consist of less than ten holdings and have a modal value of 2 holdings (Figure 1B). However, large SOAs do exist with the largest consisting of 250 holdings, although this is an outlier with the next largest consisting of 48 holdings. Holdings within SOAs tend to be located close to one another. Over 90% of the holdings that comprise each SOA are within 10 km of the other holdings of that SOA, while almost 100% are within 50 km (Figure S1), implying that intra-SOA disease spread alone will result in few, if any, long-range jumps leading to new areas of the county becoming infected.

2008 AMLS census data for England and Wales was analysed to determine the number of livestock that reside within SOAs. SOAs contain a large proportion of the livestock in England and Wales: 48.7%, 33.7% and 21.7% of sheep, cattle and pigs respectively. Most SOAs had all livestock recorded within a single holding. Every SOA consisting of 27 or more holdings had all livestock recorded within a single holding. Even those SOAs with animals recorded at more than one holding within the SOA tended to have the majority of their livestock reported at a single holding. For example, of the 345 SOAs with animals recorded at two or more holdings within the SOA, 143 had over 90% of animals at one holding. It is understandable that the June agricultural survey might record all pigs and sheep of a single owner to a single CPH, but it is not clear why cattle on CTS also tended to be reported at the same single holding, and so sensitivity to livestock locations must be considered when using these data for the purposes of disease control analyses.

**Figure 2:**
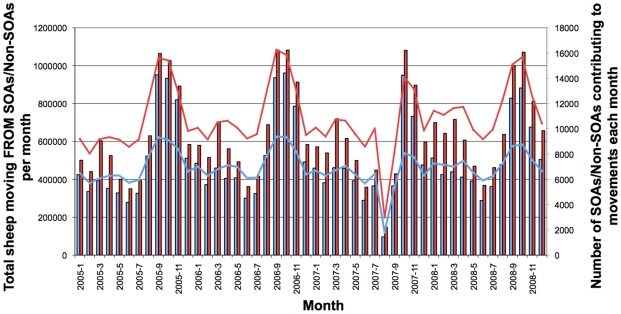
Comparison of sheep movements from SOAs vs Non-SOAs between 2005 and 2008. Monthly sheep movements from agricultural holdings within SOAs are shown in blue bars, whilst monthly sheep movements from agricultural holdings not in SOAs (Non-SOAs) are shown in red bars. The total number of SOAs and Non-SOAs contributing to the month's sheep movements are shown with the blue and red lines respectively. Movements to the same holdings or the same SOA were removed along with movements to slaughter. Only movements from “agricultural holdings” to other “agricultural holdings” or “store markets” were considered.

AMLS movements were analysed to determine how the overall volume of movements attributed to holdings within SOAs has been changing since 2005. As can be seen in Figure 2, AMLS recorded movements of sheep “from” SOAs contribute 44% of all recorded movements “from” agricultural holdings on AMLS. This is to be expected given that almost half (48.7%) of all sheep in England and Wales reside within SOAs. Movements “to” SOAs also accounted for a major percentage of the overall movements “to” agricultural holdings on AMLS (Figure S2). In both categories, there are seasonal variations in movement volumes, but overall SOA movements appear stable. Similarly, AMLS pig movements to and from SOAs were also analysed and again showed seasonal variations but overall appear stable; CTS cattle movements to and from SOAs were not analysed in this study.

There appear to be very few intra-SOA movements recorded on AMLS, only ∼500 SOAs have an intra-SOA movement recorded in 2008. This may be surprising given that all intra-SOA movements must be reported and that one of the main advantages of forming an SOA is so that animals can move freely within the SOA without observing standstills. However, this could in part be explained by the previous 5 mile rule for sheep and goats, as CPHs within SOAs tend to be located very near each other and are under the same management and control. Therefore, although the lack of reported intra-SOA movements may be expected, it does present a difficulty for modelling strategies due to the lack of data on intra-SOA movement activities. Furthermore, not many SOAs are leaving the scheme (Figure S3), and analysis showed that a substantial number (approximately one third) had no movements reported on AMLS in 2008, suggesting that many SOAs may well be redundant and are no longer being used; although these SOAs may well be active on CTS.

### Data Analysis – CTS Links

**Figure 3:**
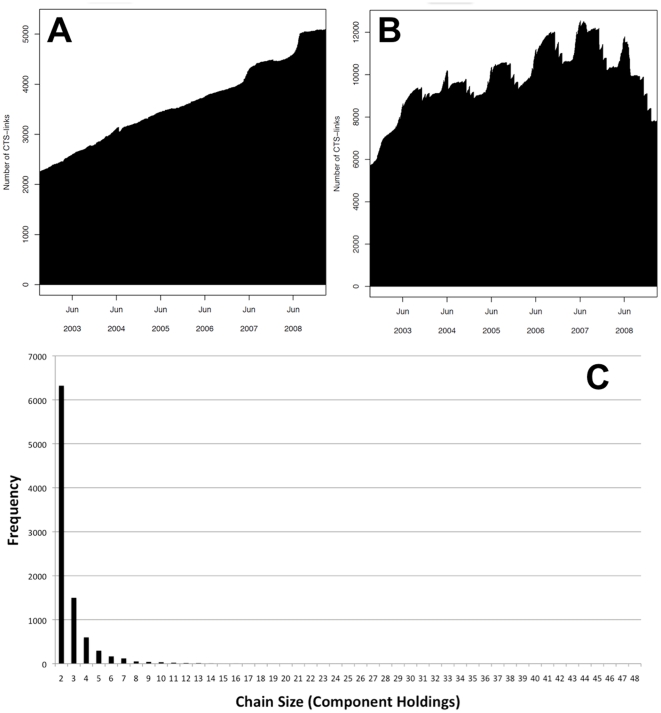
Daily total number of active CTS Links. The daily number of active CTS Links classed as Shared Facility (A) and Additional Land (B) from 2003 to 2008. (C) Shows the frequency distribution of CTS Chain sizes; the outlier chain of size 242 was removed from this chart, whilst the second largest chain consist of 48 component holdings.

Data on all active CTS Links between 2003 and 2008 in GB were combined with CTS movement data for an analysis of CTS Links and the holdings that comprise them. As can be seen in Figure 3a, the number of CTS Links classed as “Shared Facility” has increased dramatically with the number of links almost doubling between the start of 2003 and end of 2008. Furthermore, the number of links registered as “Additional Land” has also increased (Figure 3b), but shows substantial (seasonal) variation over time due to the transient nature of such links. At the start of 2008, there were approximately 17,500 active CTS Links involving just under 26,000 different holdings. Overall, the increase in linked holdings may well be a cause for concern in terms of disease spread, especially if Shared Facilities imply close and frequent contact between livestock from different holdings.

**Figure 4:**
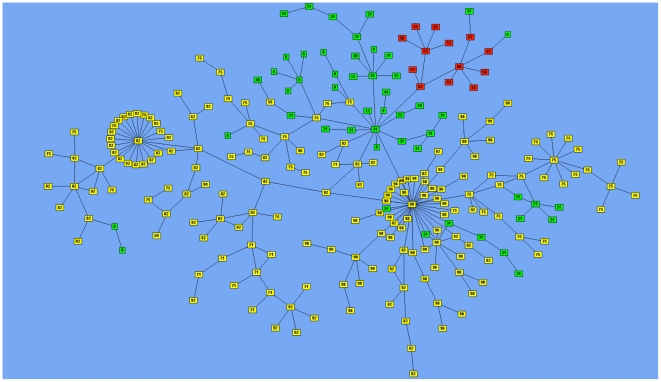
Network structure of the largest observed CTS Chain. This is the network structure of the largest CTS Chain consisting of 242 holdings. Each box represents a distinct holding, and links between boxes represent CTS Links. Boxes coloured Red, Green, and Yellow are holdings located in Wales, England, and Scotland respectively. Boxes are labelled with their holding's county number.

There is no limit to the number of CTS Links that a holding can be in, but the majority of holdings are involved in only a single CTS Link (Figure S4). However, some holdings are involved in a large number of links. As a result, groups of linked holdings can be formed from the amalgamation of these individual CTS Links, which we have termed “CTS Chains”. CTS Chains can be large (Figure 3c), with the largest chain involving 242 distinct holdings, although the next biggest chain consists of only 48 premises. While there is a 25-mile distance limit on shared facility links, there is no distance limit on additional land links so CTS Chains can be geographically dispersed. For example, the largest chain in 2008 (242 holdings) links southwest Scotland to the North of England, and onwards to North Wales (Figure 4). A chain of such a size is a clear concern, but cancelling just a few of the component CTS Links would break this chain down into smaller, less geographically dispersed units. Therefore, perhaps the resulting CTS Chains should be considered during the application process for individual CTS Links. In addition, some CTS Chains can be increased further in size when SOAs are also considered, as there are currently no restrictions on holdings being within both an SOA and a CTS Link at the same time. The structure of the largest CTS Chain (Figure 4) shows that although two of the holdings are involved in a large number of links, the majority of holdings are involved in relatively few links. This could well imply that if a disease were introduced into one end of a large chain, infection would probably take a long time to filter through the individual binary links that comprise the CTS chain to infect all the other holdings.

**Figure 5:**
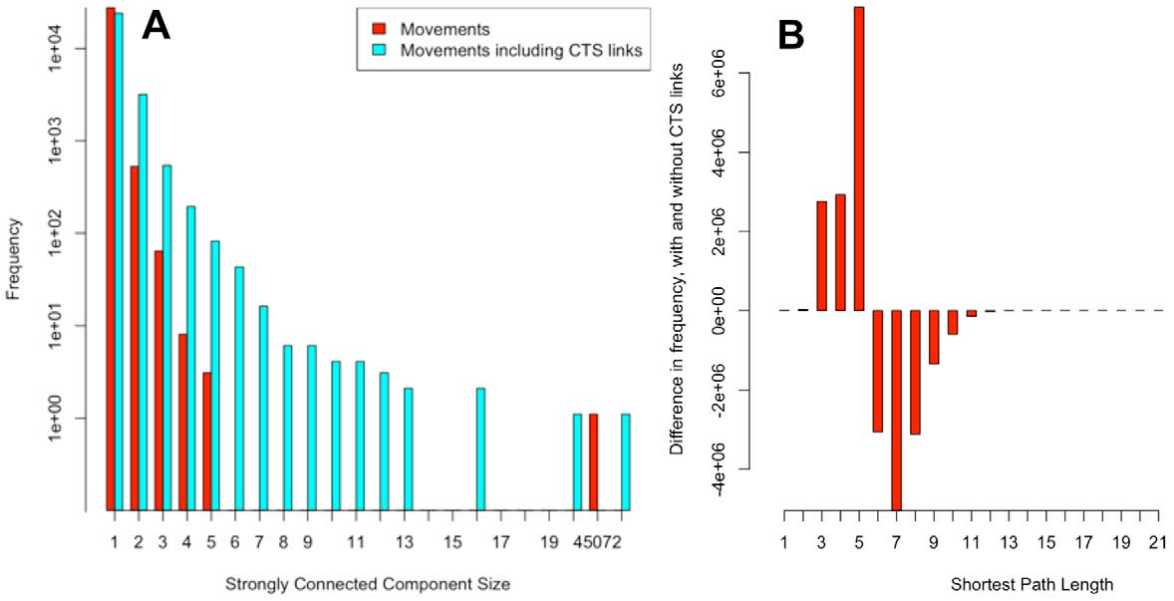
Shortest path analysis of cattle network with and without CTS Links. (A) Strongly connected component size of the cattle movements network for 2008 with (blue) and without (red) CTS Links. Adding CTS Links increases the size of the largest component (from 45072 to 54786, not shown here) and also increases the size and frequency of the smaller components; (B) Difference between the shortest path length distribution of connected premises in the cattle movements network for 2008 with and without CTS Links. The path length difference is calculated by subtracting the distribution of shortest path lengths for the movement network from the movement network with CTS Links (comparing the same connected premises).

A directed network of 73,926 premises (nodes) and 317,816 movements (edges) was created based on batched GB cattle movements for 2008. CTS Links active on 01/01/2008 consisted of 25,932 premises and 35,404 links between them. Adding these CTS Links to the network creates more connections and as expected, the movement network is, in a sense, more connected. The distribution of strongly connected component sizes (the size of the maximal set of nodes such that there is a directed path connecting each pair of nodes) for the network with and without these CTS Links (Figure 5a) shows a marked difference in structure; without CTS Links the movements network has one very large component (composed of 45,072 nodes) and most of the other premises are either unconnected or part of a small component. Adding the CTS Links, although creating a larger network of 88,654 nodes and 350,648 edges, also increases the number of nodes in the larger component to 54,786 and the frequency and size of smaller components. There is a corresponding decrease in the number of unconnected nodes once CTS Links are added.

Of the CTS Links (nodes and edges) that are added to the movement network 43.2% of the premises are already in the movement network (having had a movement of cattle in the year). It is interesting to note that 7.3% of CTS Links were already present in the movement network, although this could be a result of CTS Links that did not exist for the whole of 2008 and so movements were reported. However, if we compare the shortest paths between connected premises in the movement network before and after adding the CTS Links, (Figure 5b) we see that adding CTS Links shortens the paths between the connected premises in the movement network. Thus the CTS Links provide a mechanism for moving between premises in fewer steps.

**Figure 6:**
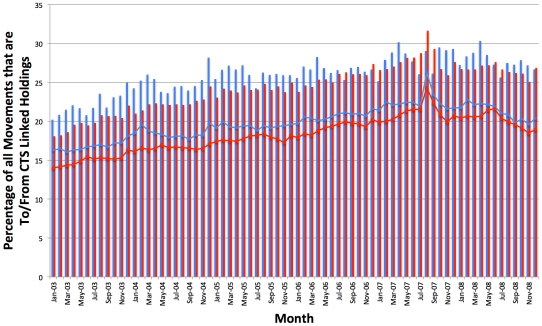
Movements to and from CTS Linked Holdings represented as a percentage of total movements. This chart shows the monthly movements on CTS that come From (red bars) and go To (blue bars) CTS Linked holdings, represented as a percentage of total movements from and to all holdings over time. For the ‘From’ category, only normal and inferred off movements from agricultural holdings in the VLA_MOVEMENTS table of CTS were considered, whilst for the ‘To’ category, only normal and inferred on movements to agricultural holdings were considered. The blue and red lines represent the percentage of holdings that have a From (blue) or To (red) movement that month and are in a CTS Link.

CTS movement data were analysed to see if the overall volume of movements attributed to holdings within CTS Links has been increasing over time. As can be seen in Figure 6, movements “to” agricultural holdings in CTS Links (represented as a percentage of movements “to” all agricultural holdings) has been steadily increasing over the years from 20% in 2003, peaking at 30% in mid 2007, and finishing at 27% at the end of 2008, as would be expected from the increase in holdings registered with CTS Links. Movements “from” agricultural holdings in CTS Links also show a similar pattern (Figure 6). The proportionate increase in movements from CTS Linked holdings in August 2007 (Figure 6) represents their greater proportion of movements to slaughter, which were largely unaffected at a time when other movements were being restricted due to the outbreak of FMD at that time.

### Modelling - SOAs

In the 2001 FMD epidemic, two factors resulted in the exceptional difficulties associated with control. First, the rapid rise in numbers of holdings affected meant that logistical capability was strained to capacity and beyond, and second, the wide geographical extent meant that the control effort could not be concentrated in one area, further increasing the cost of control [9]. Here, we investigate the impact of exemptions on both the number of premises affected, and the geographical distribution.

Because of the potentially complicated interactions between spatial spread, spread due to different species and spread due to the dynamic network of livestock movements, we use a simulation approach to summarise the impact of all these factors on the two outcomes described above – number of premises and geographical spread. In our previous work [3], CTS and AMLS movement data were used to construct an individual holding based model of the initial spread of FMD in GB to determine the susceptibility of the GB livestock industry to future outbreaks. Transmission through movements was modelled, with additional local spread unrelated to the known movements, as well as intra-SOA spread. Simulations showed that movements can result in a large nationwide epidemic, but only if cattle are heavily involved, or the epidemic occurs in late summer or early autumn. Inclusion of local spread can considerably increase epidemic size, but has only a small impact on the spatial extent of the disease. Importantly, the inclusion of intra-SOA spread had only a minimal impact on final epidemic size. However, the increased number of SOAs since then and the additional effect of CTS Links merits further investigation of their impact on FMD spread.

Therefore, this approach [3] was updated to consider the updated data on both SOAs and CTS Links and was re-run with the latest movement data from AMLS and CTS for 2008; the model was run for England and Wales only, as updated Scottish data were unavailable at the time of the analysis. We also consider two stocking scenarios for intra-SOA spread: (1) where the recorded distribution of stock numbers within SOA member holdings is correct; and (2) where stock are evenly distributed amongst SOA member holdings. While the mechanisms of SOAs and CTS Links are different, for this study we assume that their role in FMD spread is similar – rapid spread amongst holdings that would otherwise be of limited risk to each other, thereby increasing the potential for onward spread to other holdings. Although this is a worst-case scenario, there is currently insufficient information on SOA stock levels as well as intra-SOA and intra-Chain movements to develop realistic alternatives.

We allow the epidemic to run for a period of one month, in order to explore the complex relationship between the explicit network of livestock movements, and the possible role of dispensations on disease spread. This is not meant to be realistic; identification of FMD on a holding would result in an immediate movement ban, possibly at the national level, and the probability of detection is related to a number of factors, most importantly the number of premises affected. However, use of a fixed time frame for spread provides for a basis of comparison with our previous work [5] and is an indication of the relative impact of these dispensations on FMD spread, compared to 2001 when silent spread occurred for roughly this period.

**Figure 7:**
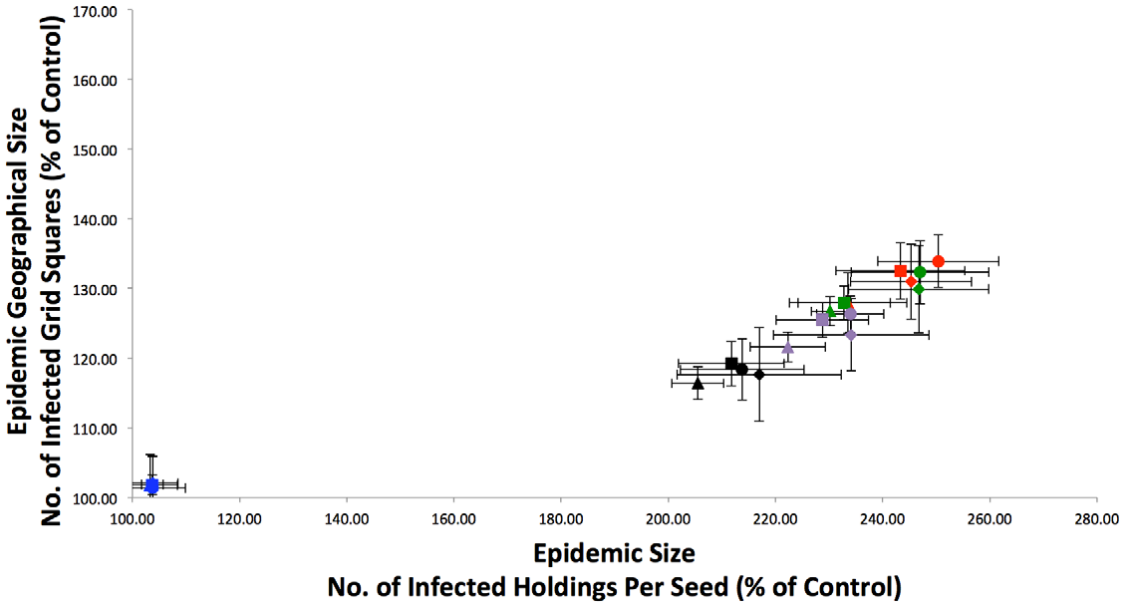
Comparison of epidemic sizes with SOAs. The model was run including SOAs, with no stock restriction on intra-SOA spread and the following distance limits on intra-SOA spread: No Limit (red), 50 km (green), 16 km (purple), and 8 km (black). In addition, the results from the SOA model with intra-SOA spread restricted to holdings with stock recorded as present is shown in blue. Epidemic size (number of infected holdings per seed) and geographical size (number of infected grid squares per seed) were firstly transformed into percentages of the control epidemic size (with no linked holdings) from the same time of year. Secondly, averages were then obtained for the four quarters of the year (Jan-Feb-Mar[squares], Apr-May-Jun [circles], Jul-Aug-Sep [triangles], Oct-Nov-Dec [diamonds]) giving four data points for each scenario. Error bars are associated with each quarterly average value that represent the 95% confidence intervals for that quarter, assuming a normal distribution and a standard deviation calculated from that quarter's data.

As can be seen in Figure 7, when intra-SOA spread is limited to only holdings with reported stock, the inclusion of SOAs appears to have little effect on epidemic size. This is consistent with our previous results [3] which did not investigate alternative stocking scenarios. As the majority of SOAs have reported stock at only one holding within the SOA, there is little intra-SOA spread recorded in the model as there are no other stock containing holdings within the SOA to infect with the disease and subsequently spread out from via local spread.

**Figure 8:**
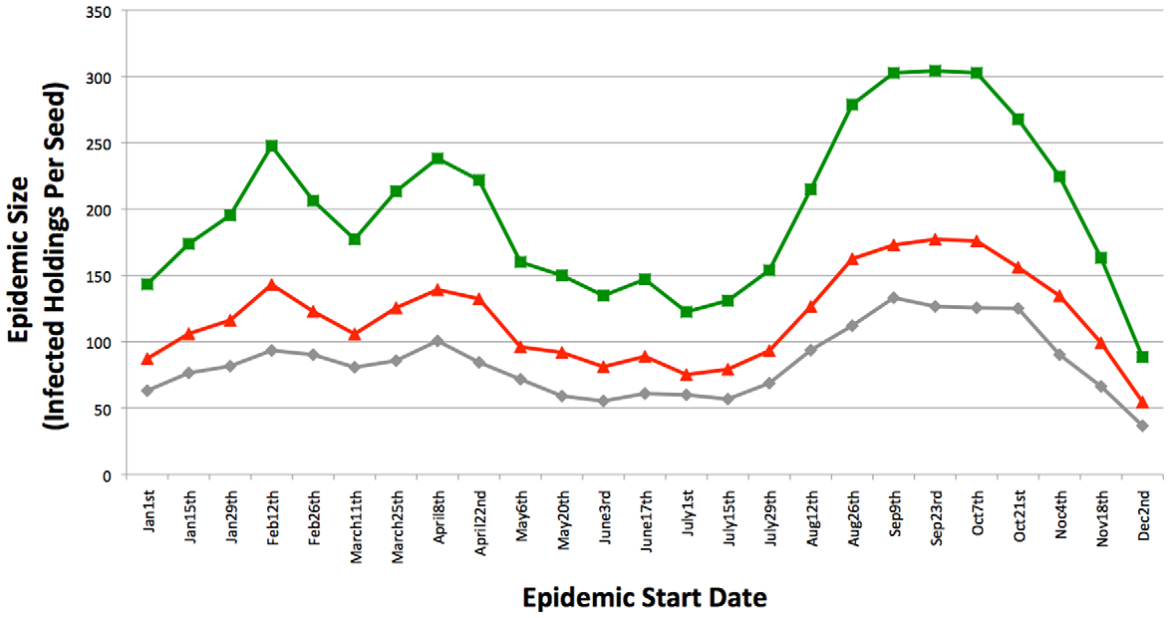
The effect of SOAs on epidemic size – source of infection. Each data point is the number of infected holdings per seed, generated from 400 stochastic simulations of FMD epidemics starting at that time of year, each with five seeds. The model keeps track of how each holding becomes infected, which can be via one of three routes: 1) Movements; 2) Local spread; and 3) Intra-SOA spread. The grey line shows the total number of infected holdings per seed for the normal Control model, with no SOAs or linked holdings. The grey line is therefore composed of Movement and Local spread infection events only. The red line shows the number of Movement and Local spread infection events (per seed) for the SOA model, which increases the size of epidemics throughout the year. However, the green line shows all (Movements+Local+intra-SOA) infection events (per seed) for the SOA model. As can be seen, the biggest contributor to the increase in epidemic size as a result of including SOAs is intra-SOA spread itself– other premises within the SOA becoming infected. Epidemic sizes are measured in terms of number of infected holdings – not number of infected animals.

However, when this stock limitation is removed and one assumes animals are evenly distributed throughout the SOA, bigger epidemics occur throughout the year (Figure 7). Note however that epidemics are larger in terms of holdings infected, rather than animals infected, and that the within SOA animal population is the same but distributed among more holdings. The model was used to investigate whether imposing distance limits on the holdings that comprise the SOAs would have an affect on epidemic size. Distance limits of 50 km and 16 km have little effect on epidemic size, whilst 8 km has only a small effect (Figure 7), consistent with the usual close proximity of holdings within SOAs. This suggests that the majority of the effect of increased epidemic size is the “book-keeping” increase in infected holdings within SOAs themselves, rather than new areas being affected. Indeed, as the model categorises the cause (movement, local, or intra-SOA spread) of each infection event, one can see that the increase in epidemic size is predominantly the result of holdings becoming infected via intra-SOA infection spread (Figure 8). However, infection via movement and local spread does also increase suggesting there is also onward transmission from other holdings within the SOA.

Another measure of risk is the geographical spread of the epidemic. In the model, GB is subdivided into a grid consisting of squares of 100 km^2^, where each square contains the relevant holdings as defined by their easting/northing coordinates. The number of grid squares with at least one infected holding at the end of the simulation is a proxy for the extent of geographical spread. The imposition of distance limits to constrain intra-SOA spread has little effect on geographical spread (Figure 7), indicating that long range jumps infecting new parts of the country are rare, and that the increased geographical spread is predominately local with neighbouring grid squares becoming infected. Therefore, under the worst-case scenario (one infected – all infected) of intra-SOA spread, although an increase in logistical (veterinary, slaughter teams etc.) resources may likely be required to handle any increase in the number of infected premises, these resources would not be required in a much larger geographical area as a result of intra-SOA spread.

### Modelling – CTS Links

**Figure 9:**
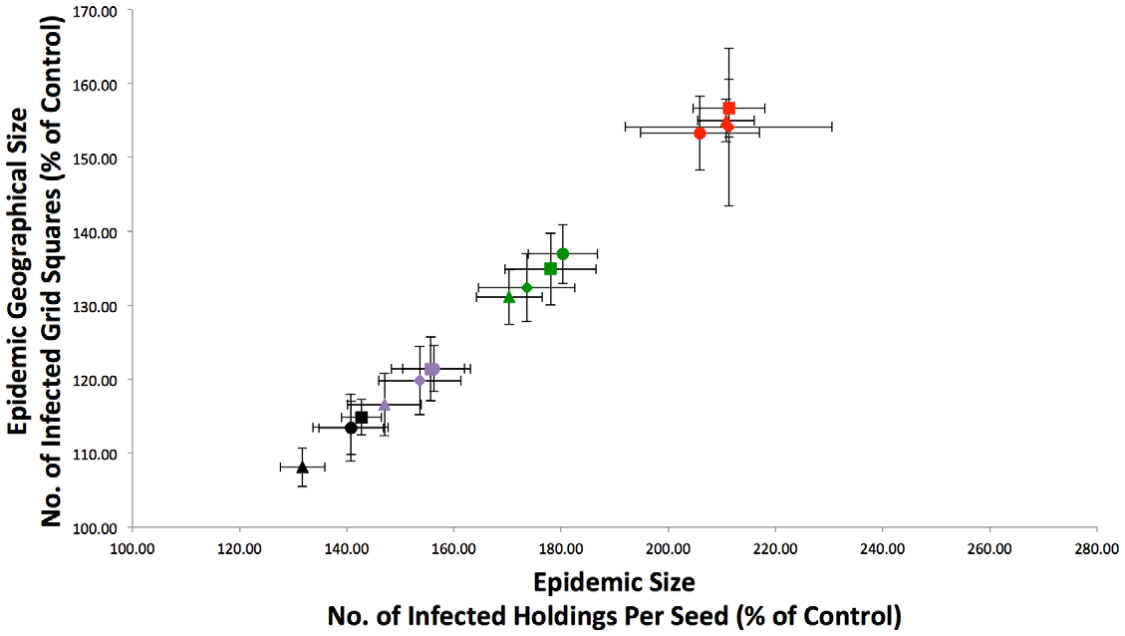
Comparison of epidemic sizes and geographical spread with different linked holdings. The model was run including CTS Chains, with the following distance limits on intra-Chain spread: No Limit (red), 50 km (green), 16 km (purple), and 8 km (black). Epidemic size (number of infected holdings per seed) and geographical size (number of infected grid squares per seed) were firstly transformed into percentages of the control epidemic size from the same time of year. Secondly, averages were then obtained for the four quarters of the year (Jan-Feb-Mar [squares], Apr-May-Jun [circles], Jul-Aug-Sep [triangles], Oct-Nov-Dec [diamonds]) giving four data points for each scenario. Error bars are associated with each quarterly average value that represent the 95% confidence intervals for that quarter, assuming a normal distribution and a standard deviation calculated from that quarter's data.

Although CTS Links form dyads, numerous holdings are involved in more than one link. Therefore, CTS Links can be amalgamated together to form CTS Chains, which can be imported into the model and treated as sets of holdings equivalent to SOAs. The inclusion of CTS Chains alone (without SOAs) into the model does substantially increase epidemic size (Figure 9), although CTS Chains have less of an effect on epidemic size than SOAs. Furthermore, CTS Chains substantially increase the geographical spread of the epidemic (Figure 9), resulting in smaller but more geographically dispersed epidemics when compared to SOAs. As in the SOA model, we then applied distance limits to intra-CTS Chain spread of 50 km, 16 km, and 8 km (Figure 9). In contrast to SOAs, the distance limits are important, with the 50 km limit having a large impact on epidemic size, and the 16 km and 8 km limits reduce epidemic sizes still further (Figure 9). However, increased epidemic sizes are still observed even with severe distance limits. The geographical spread of epidemics when CTS Chains are incorporated into the model was also examined. The distance limits again have more of an effect on geographical spread when compared to SOAs (Figure 9), with the 8 km limit reducing the geographical spread to close to that observed in the control model with no linked holdings at all. This again highlights the fact that holdings linked via CTS Links are more geographically dispersed than those linked via SOAs.

### Modelling – SOAs and CTS Links

SOAs and CTS Links/Chains can be combined together to form a single set of linked holdings, resulting in even bigger epidemics than SOAs or CTS Links alone (Figure S5).

## Discussion

Although there was an initial rapid increase in the number of SOAs when they were introduced, their numbers have only been increasing gradually in recent years. Furthermore, the volume of movements attributed to SOAs over recent years remains relatively constant. However, the analysis did reveal a number of points of potential concern. There appear to be very few intra-SOA movements recorded in the AMLS database despite a requirement that all such movements be reported. This could be explained by the previous 5 mile rule for sheep and goats, whereby movements under 5 miles between land under the same ownership and control did not need to be reported. As holdings within SOAs tend to be located very near each other, and are under the same control, this could well explain the lack of intra-SOA movements; we note that the 5 mile rule for sheep and goats has recently been disbanded [11] and now all movements between different CPHs must be reported. An alternative explanation for the lack of intra-SOA movement data is that there could be confusion on the reporting requirements with different rules governing SOAs and CTS Links combined with the 5 mile rule for sheep and goats.

Our modelling work showed that SOAs do pose a potential risk of increasing the number of holdings infected in an epidemic considerably, if the worst case scenario of “one infected – all infected” is assumed. However, the majority of the effect of increased epidemic size is the “book-keeping” increase in infected holdings within SOAs themselves. Furthermore, SOAs did not greatly effect the geographical spread of the epidemic, which is to be expected given that the component holdings within an SOA appear to be located near each other, typically within 10 km. Overall, the effect in terms of epidemic size of including SOAs into simulations appears to be mainly due to intra-SOA spread, in that infection events as a result of the disease spreading within the SOA contributes a large proportion to the overall epidemic size. There is currently insufficient information on stock levels and intra-SOA movements to determine whether or not this worst case is relevant, and so it is an indicator of a need for future data collection.

If the reported stock levels are correct, in that typically an SOA only has stock located at one holding, then SOAs would not appear to be a major risk in terms of epidemic size, as holdings without stock can not contract and therefore pass on the disease via local and movement based spread. This is consistent with additional registered CPHs within an SOA representing grazing land with no permanent stock. For example, within an SOA of five holdings that are used for grazing, at any one time only one holding may have stock on it, thus limiting the possibilities for disease spread. However, Figure 7 shows that there is a considerable difference if premises are stocked more evenly, suggesting that a more detailed understanding of intra-SOA movements and stock levels would be useful. The simulation results presented here are in terms of infected holdings, whereby intra-SOA spread results in more holdings becoming infected. However, if the majority of SOA holdings have no stock, then the number of livestock slaughtered within the SOA will increase little as a result of intra-SOA spread. It should also be noted that all valid SOAs are included in the above simulations. The data analysis did suggest that there could be a redundant accumulation of SOAs, as few SOAs seem to leave the scheme, and a fair amount of SOAs appear to be inactive in terms of movement into or out of the SOA on AMLS. Given the potential risk of SOAs in Figure 7, this could suggest that a clean up of inactive SOAs would be valuable in assessing the true risk associated with SOAs.

The number of CTS Linked holdings, especially those linked via Shared Facilities, appears to be increasing rapidly and the volume of movements from such CTS linked holdings is also increasing. This is in contrast to AMLS movements from SOAs, which remained relatively constant over time, and suggests that there is little redundant accumulation of inactive holdings within the CTS Links. Currently, there is neither a limit on the number of CTS Links an individual holding can be in, nor a limit on the distance between CTS linked holdings via the Additional Land category. Combined, these features can lead to large chains of linked holdings that can also be geographically dispersed throughout the country; however, the vast majority of chains only contain 2 holdings. These chains can then grow even further as there is currently no restriction on being within an SOA and in CTS Links at the same time.

In the worst case scenario (all members of a CTS Chain potentially directly linked, or linked within a short time), large and geographically dispersed CTS Chains are potentially a risk in terms of disease spread. Applying a 50 km distance limit on transmission amongst CTS Links markedly reduces epidemic size, suggesting that allowing long-range links or a short series of links covering a large distance may be an important risk. However, further investigations should be made as to whether or not the CTS Chains are also transmission chains – if multiple links in a chain are not used in the timeframe of FMD spread (at most a few weeks for single holdings), then they are not important in this context. Even if all links in the chain were active, it would take time for the disease to spread via cattle movements through each binary link in the largest chain – this was not considered here as we used a “one infected – all infected” approach to intra-CTS Chain spread similar to intra-SOA spread. Furthermore, while movements are not recorded, standstills are still expected to be in effect, and investigation into standstill compliance within chains may be merited.

The scenarios presented here examine worst-case scenarios for disease spread, with members of SOAs assumed to have the same infection status throughout. In reality, this would only be the case in a limited number of instances in a real outbreak, and although our data analysis does highlight a number of points for further investigation, it is important to balance the need for adequate disease prevention and control with commercial activity so that holdings can perform competitively. Shared Facility links where holdings share milking parlours are clearly essential, and would likely involve neighbouring farms. In terms of FMD spread, if one holding were to become infected, any neighbour would be at high risk of infection, irrespective of whether an SOA or CTS Link were present. One could question the need to link two holdings that are very geographically dispersed, as it would be unlikely that cattle movements between the two holdings would be frequent. Therefore, distance limits on Additional Land links may well be considered. We note that there was previously a 50 km distance limit between holdings within an SOA, although this has now been disbanded. Furthermore, a limit on the number of CTS Links that an individual holding can be in may well reduce the observed CTS Chains to smaller less risky units. The increase in the use of CTS Links as well as SOAs could simply reflect changes within the farming industry. If there are fewer farms, those that remain may become bigger through expansion into old farm land, but distinct CPHs must still remain due to distance rules on how far farm land can be from the main steading.

Broadly speaking, our analyses suggest that while SOAs and CTS Links are important, they are important because of the number of holdings so registered, rather than because of the activity associated with them. Under a worst case scenario of “one infected – all infected” both SOAs and CTS Links pose a risk of increasing epidemic sizes, but further investigations are needed into whether CTS Chains are transmission chains, and also investigations into intra-SOA movements and livestock distributions, in order to develop realistic alternative scenarios.

## Methods

### Data

Full details on the different data sets used in the data analysis and model simulations can be found in the Text S1. For the modelling, livestock movement data for England and Wales in 2008 from both CTS and AMLS was used. Movements to slaughter were removed. CTS movements were batched, where individual movements with the same dates, departure and destination holdings were grouped together; AMLS data is already in a batch format. Holding location data (such as CPH, holding type and easting/northing co-ordinates) also came from AMLS and CTS whilst holding population data came from the 2008 census.

### Model

The model [3] was individual-based at the level of the holding, and stochastic. Holdings were described by their dates of becoming exposed, infectious via movements, infectious via local spread, and removed, determined by the timings of movements and local spread events. Based on these dates, the holdings could be classed into one of the four states:

- **S** - These are holdings without exposed or infectious animals.
- **H -** These are farms containing animals exposed to infection, but with all these animals subject to isolation (triggered by the movement of animals onto the farm) until the movement restriction period has elapsed. The movement restriction period is 20 days for pigs, whereas for sheep and cattle, it is 6 days for England and Wales, and 13 days for Scotland [8]. These holdings do not constitute a source of further infection by either movement or local spread.
- **E -** These holdings are similar to H, except with exposed animals not under movement restriction (e.g. it was infected by local spread), thus constituting a risk of further infection through off movements. These holdings are not yet infectious by local spread, but off-movements can carry exposed animals. A latent period of 3 days was used, within the range given by [6].
- **I -** These are holdings containing infectious animals, after the latent period, which are a source of infection by both off-movement and local spread. FMD can spread rapidly within a population, infecting whole herds of cattle or pigs within one cycle of infection (ca 3 days). Therefore, we consider the entire holding as potentially infectious within this period after exposure [13].

Once infected, farms are assumed to remain infectious until the end of the simulation. Markets, however, are assumed to be disinfected and not continually occupied by livestock. Therefore in the model, infected markets re-enter the S state at the end of the following day, to allow for single overnight stays of livestock.

Epidemics were seeded by selecting a fixed small number of holdings (5 seeds are selected) from those with off-movements on the first day of the simulation. These are set to be infectious on this day. Consistent with the 2001 epidemic (e.g. [6]), the simulations were run for 28 days, beyond which it is assumed unlikely that an epidemic could persist without being identified. For the Pan Asia strain of FMD, clinical signs in cattle and pigs are typically detected quickly; it is more difficult to detect infection in sheep, but these are also less susceptible [14].

If a holding is exposed or infected, there are three ways in which the disease can spread to other holdings:

1. **Movements:** Only movements from exposed (E) or infected (I) holdings or markets can cause infection, and the risk of infection is assumed higher for movements of large numbers of animals. Different probabilities of infection, which are then weighted by the number of animals moved, are assumed for the movements of sheep compared to movements of cattle or pigs; movements of cattle or pigs from an infected holding are all assumed to be potentially infectious, whilst movements of sheep from an infected holding have a lower probability of infection. Furthermore, different probabilities of infection are assumed for movements from an infected market, as such movements are less certain to be infectious than those from an infected holding. Thus, for movements off a market (in the E or I state), μ was set at μ_m_sheep_ = 0.004 for sheep and μ_m_other_ = 0.02 for cattle and pigs. For other movements, we assumed a value of μ_other_ = 1.0 for cattle and pigs (all movements are potentially infectious) and μ_sheep_ = 0.02 for sheep. These movement parameters were based on the 2001 FMD epidemic and analysis of movements from infected holdings that resulted in infection spread [3].
2. **Local Spread:** Local spread unrelated to known movements from infected (I) holdings was modelled using a constant rate of generation of new infected holdings per day per infected holding β = 0.065. Local spread is assumed to be a combination of factors, including human and vehicle movements, possible airborne spread and nose-to-nose contact of livestock across boundaries. On each simulation day, a number of infectious contacts were selected for each infectious holding from a Poisson distribution with mean β, without replacement, considering only holdings reported as containing livestock in the agricultural census (or redistributed SOA population). Contacts were limited to a 10 km radius, and weighted according to distance d by p∼e^−ad^, where a = 0.5 km^−1^. These local parameters were based on 2001 FMD epidemic sizes [3], [15]. Susceptible contacts become exposed on the current day of simulation. Infection by local spread negated the effect of any imposed standstill, and off movements from infected holdings were considered to potentially contain exposed animals.
3. **Intra-SOA spread:** Although movements within an SOA now need to be reported, very few within-SOA movements are recorded. This could be due to confusion on reporting requirements. Therefore, if one member of an SOA becomes infected, we assume that the rest may be infected too, due to unrecorded movements of infectious animals within the SOA. Upon infection of a holding on a given day, the model (optionally) identifies any other holdings within the same SOA, these then become infected and gain the same date of exposure as the source holding, but only become a source of further infection on the subsequent day (the day after the initial holding in SOA became infected), to allow time for distribution of animals within the SOA. In addition to the above, “one infected – all infected” option for within SOA spread, two other options for intra-SOA spread are available. In the model, all SOAs that were active on 01/01/2008 were considered in simulations throughout 2008. For CTS Links, all links that were active on 01/01/2008 were considered. These were then amalgamated into CTS Chains of linked holdings to form SOA like groups to create a worse case scenario of one infected – all infected in the CTS Chain. Three linked holding scenarios were considered in the model: (1) SOAs only; (2) CTS Links only, and (3) SOAs and CTS Links.

Epidemic simulations were run with starting times varied across the 2008 at 14-day intervals and repeated simulation at each starting point (simulations were repeated 400 times). The exact start day used in each simulation was subject to up to 14 days of jitter (i.e. a random value of up to 14 days is added to the defined start day to examine that period of the year rather than that specific day), in order to avoid unusual properties that might be associated with a single day. The distribution and prevalence of each 28-day simulated epidemic were recorded and divided by the number of seeds used.

### Network Analysis

For the network analysis, a directed network of 73,926 nodes (premises) and 317,816 directed edges (movements) were created from the GB cattle movements for 2008, excluding movements to slaughter; markets were considered for this analysis and treated as farms. The shortest path between all pairs of connected nodes was then calculated, saving the source and destination nodes of the path. CTS Links active on 01/01/2008 were then added to the network as bi-directional edges, increasing the number of nodes and edges in the network by 20% and 4% respectively. The shortest path length between the saved pairs of source/destination nodes was then re-calculated in this expanded network.

## Supporting Information

Text S1 **Model Data.** Full details on the different data sets used in the data analysis and model simulations.
(DOC)

Figure S1 **Cumulating distribution of the mean inter-farm distances of the component premises of SOAs.** Over 90% of the farms that comprise each SOA are within 10 km of the other units of that SOA, that figure is almost 100% when a distance of 50 km is studied.
(TIFF)

Figure S2 **Comparison of sheep movements TO SOAs Vs NonSOAs.** Monthly sheep movements to agricultural holdings within SOAs are shown in blue bars, whilst monthly sheep movements to agricultural holdings not in SOAs (Non-SOAs) are shown in red bars. The total number of SOAs and Non-SOAs contributing to the month's sheep movements are shown with the blue and red lines respectively. Movements to the same holdings or the same SOA were removed along with movements to slaughter. Only movements from “agricultural holdings” or “store markets” to other “agricultural holdings” were considered.
(TIFF)

Figure S3 **Number of new SOAs by month (black lines) and number leaving the scheme (red lines) by month.** After the initial peak in new SOAs at their inception, the number of farms joining the scheme each month has been marginally greater than the number leaving the scheme.
(TIFF)

Figure S4 **The number of premises to which each main holding is linked.** The majority of main holdings only have one linked premises. Links that expire and are subsequently renewed are only counted once.
(TIFF)

Figure S5 **Comparison of epidemic sizes and geographical spread with SOAs and CTS Chains combined.** SOAs and CTS Chains were combined to form one large set of SOA/Chain linked holdings. The model was run including the combined SOAs/Chains, with the following distance limits on intra-Chain spread: No Limit (red), 50 km (green), 16 km (purple), and 8 km (black). Epidemic size (number of infected holdings per seed) and geographical size (number of infected grid squares per seed) were firstly transformed into percentages of the control epidemic size from the same time of year. Secondly, averages were then obtained for the four quarters of the year (Jan-Feb-Mar [squares], Apr-May-Jun [circles], Jul-Aug-Sep [triangles], Oct-Nov-Dec [diamonds]) giving four data points for each scenario. Error bars are associated with each quarterly average value that represent the 95% confidence intervals for that quarter, assuming a normal distribution and a standard deviation calculated from that quarter's data.
(TIFF)
