# Supplementary material for: Risk of Foot-and-Mouth Disease Spread Due to Sole Occupancy Authorities and Linked Cattle Holdings
Source: PLoS One. 2012 Apr 19;7(4):e35089. doi: 10.1371/journal.pone.0035089 (PMC3331861; doi:10.1371/journal.pone.0035089)
Supplement: Text S1 — Model Data. Full details on the different data sets used in the data analysis and model simulations. (DOC) [file pone.0035089.s001.doc]

**Risk of FMD Spread due to Sole Occupancy Authorities and linked cattle holdings**

**Supporting Information Text S1**

**Data**

Information on the different data sets utilised and how they were processed for the model is presented below.

1. **AMLS** – Movement and holding data from 2003 to August 2009 was obtained from the Animal Movements License System (AMLS).
2. **SOAs** – Data (SOA ID number, holding ID number, in date, out date) on all previous and existing SOAs involving any holding in England/Wales was obtained from AMLS. There were a total of 132,205 entries in this data set.
3. **CTS** – A download of the Cattle Tracing System (CTS) database containing cattle movement (including the VLA_MOVEMENTS table) and holding data was obtained in August 2009 from the Veterinary Laboratory Agency.
4. **CTS Links** – Data (main holding (MH) cph, MH start date, MH end date, MH type, linked holding (LH) cph, LH start date, LH end date, LH holding type, cts link type) on all CTS Links in Great Britain that were open since 2003 were obtained from British Cattle Movement Service (BCMS). There were a total of 80,144 links in this data set.
5. **Census Data** – Census data comprising cattle, sheep, pig, goat and deer numbers for all holdings in England/Wales for June 2008 was obtained from AMLS and CTS.

Scottish Animal Movements Unit (SAMU) sheep/pig movement data was unavailable.

The data analysis of SOAs is for England/Wales only – AMLS data combined with SOAs involving any holding in England/Wales.

The data analysis of CTS Links/Chains is for the whole of GB – as both CTS and the CTS Links data cover the whole of GB.

The modelling work is for England/Wales only as there was no SAMU data available, Scottish holdings and Scottish cattle movements were filtered out.

**Model**

**Holdings:** The model imports a file containing details (cph, holding type, easting, northing, cattle, pigs, sheep, soa number) on each holding to be considered. This file was generated by combining the holding data from CTS and AMLS for all holdings with 11 digit CPH numbers (e.g. 01/001/0001) in England/Wales. A total of 576, 911 holdings were therefore identified. If a holding was duplicated in both data sets, the information on CTS was used by default. However, easting and northing data from AMLS was used for holdings on CTS with no co-ordinate data. On importation, the model calculates the mean easting and northing co-ordinates for each county/parish, and then checks the co-ordinates of each holding and relocates any that are unrepresentative (more than 30km away) of their county/parish mean to the mean co-ordinates of the county/parish plus jitter within 1km^2^: 2.5% of holdings were relocated in this way.

**Movements:** The model imports a file containing details (date, from holding, to holding, species, number of animals moved) on each movement to be considered. Sheep and pig movements (which are already batched) for 2007 and 2008 were extracted from AMLS; movements to slaughter were not considered, nor were movements from and to the same holding. Cattle movements for 2007 and 2008 were extracted from the CTS VLA_MOVEMENTS table. Movement to or from Scotland, movements to slaughter, and movements from and to the same holding were all removed. The remaining CTS movements were then batched (where individual movements with the same dates, departure and destination holdings were grouped together), and inserted into the AMLS movements file to create a single batched movement file which contained 3,309,976 batched movements.

**SOAs:** SOAs active on 01/01/2008 were considered in the model simulations throughout 2008. Each SOA has a unique identifier number. This number was added to each of the SOA’s component holdings in the model Holdings file.

**CTS Links/Chains:** CTS Links that were active on 01/01/2008 were processed and amalgamated to form CTS Chains, which were then used in model simulations throughout 2008. Each CTS Chain was assigned a unique number. This number was added to each of the Chain’s component holdings in the model Holdings file.
