# Supplementary figures and images for: Risk of Foot-and-Mouth Disease Spread Due to Sole Occupancy Authorities and Linked Cattle Holdings
Source: PLoS One. 2012 Apr 19;7(4):e35089. doi: 10.1371/journal.pone.0035089 (PMC3331861; doi:10.1371/journal.pone.0035089)

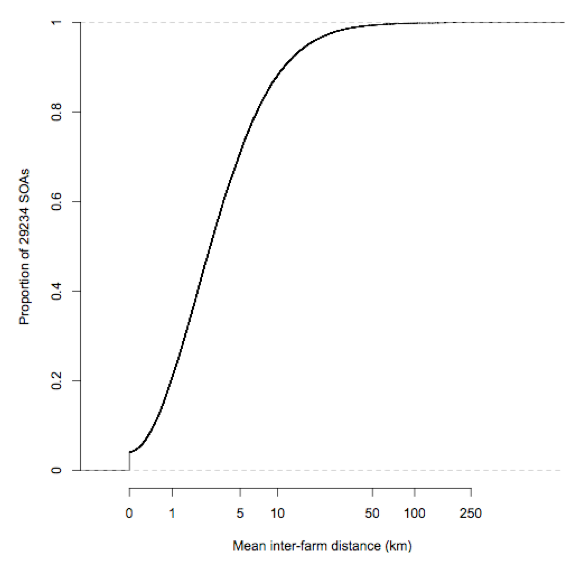

Supplement: Figure S1 — Cumulating distribution of the mean inter-farm distances of the component premises of SOAs. Over 90% of the farms that comprise each SOA are within 10 km of the other units of that SOA, that figure is almost 100% when a distance of 50 km is studied. (TIFF) [file pone.0035089.s002.tiff]

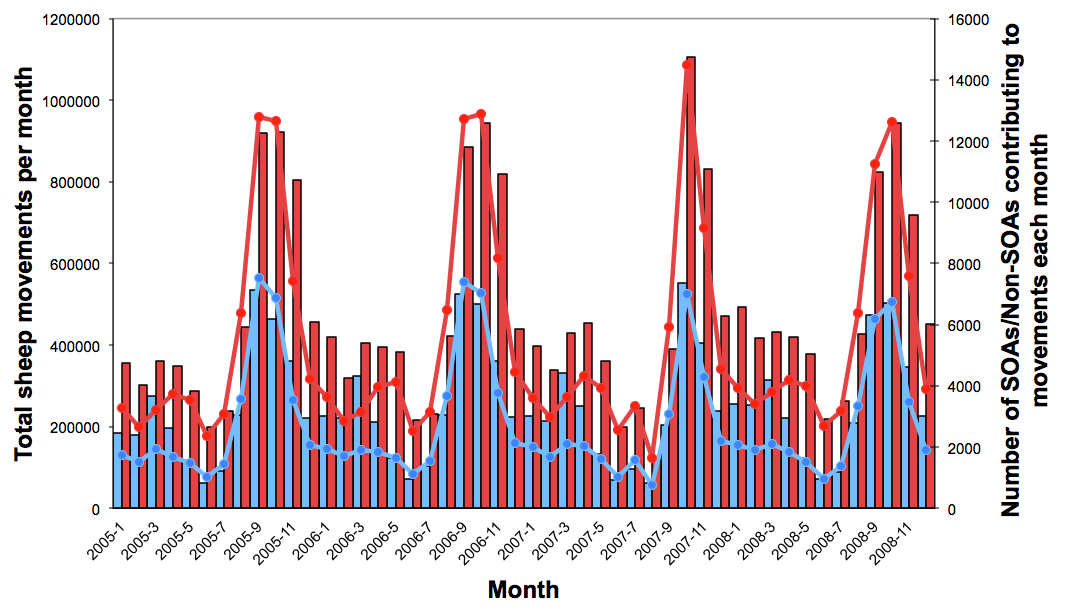

Supplement: Figure S2 — Comparison of sheep movements TO SOAs Vs NonSOAs. Monthly sheep movements to agricultural holdings within SOAs are shown in blue bars, whilst monthly sheep movements to agricultural holdings not in SOAs (Non-SOAs) are shown in red bars. The total number of SOAs and Non-SOAs contributing to the month's sheep movements are shown with the blue and red lines respectively. Movements to the same holdings or the same SOA were removed along with movements to slaughter. Only movements from “agricultural holdings” or “store markets” to other “agricultural holdings” were considered. (TIFF) [file pone.0035089.s003.tiff]

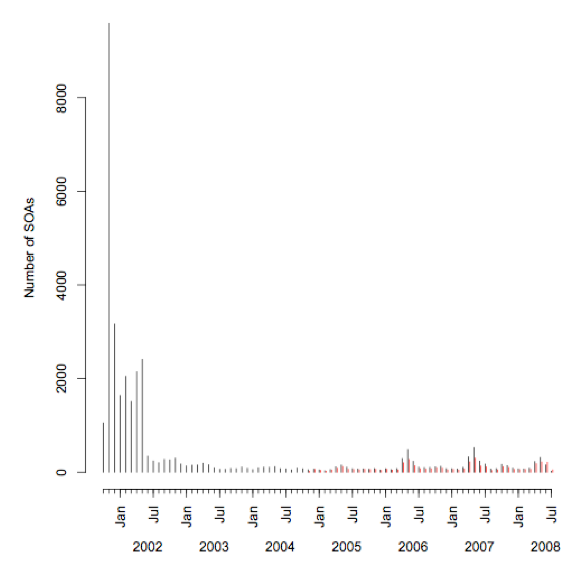

Supplement: Figure S3 — Number of new SOAs by month (black lines) and number leaving the scheme (red lines) by month. After the initial peak in new SOAs at their inception, the number of farms joining the scheme each month has been marginally greater than the number leaving the scheme. (TIFF) [file pone.0035089.s004.tiff]

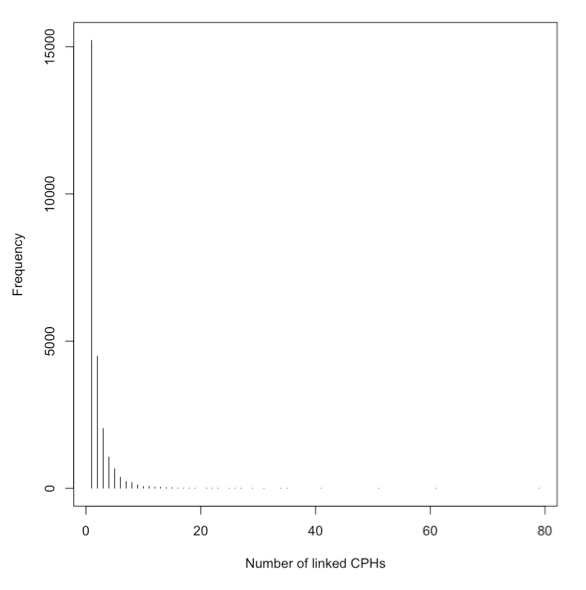

Supplement: Figure S4 — The number of premises to which each main holding is linked. The majority of main holdings only have one linked premises. Links that expire and are subsequently renewed are only counted once. (TIFF) [file pone.0035089.s005.tiff]

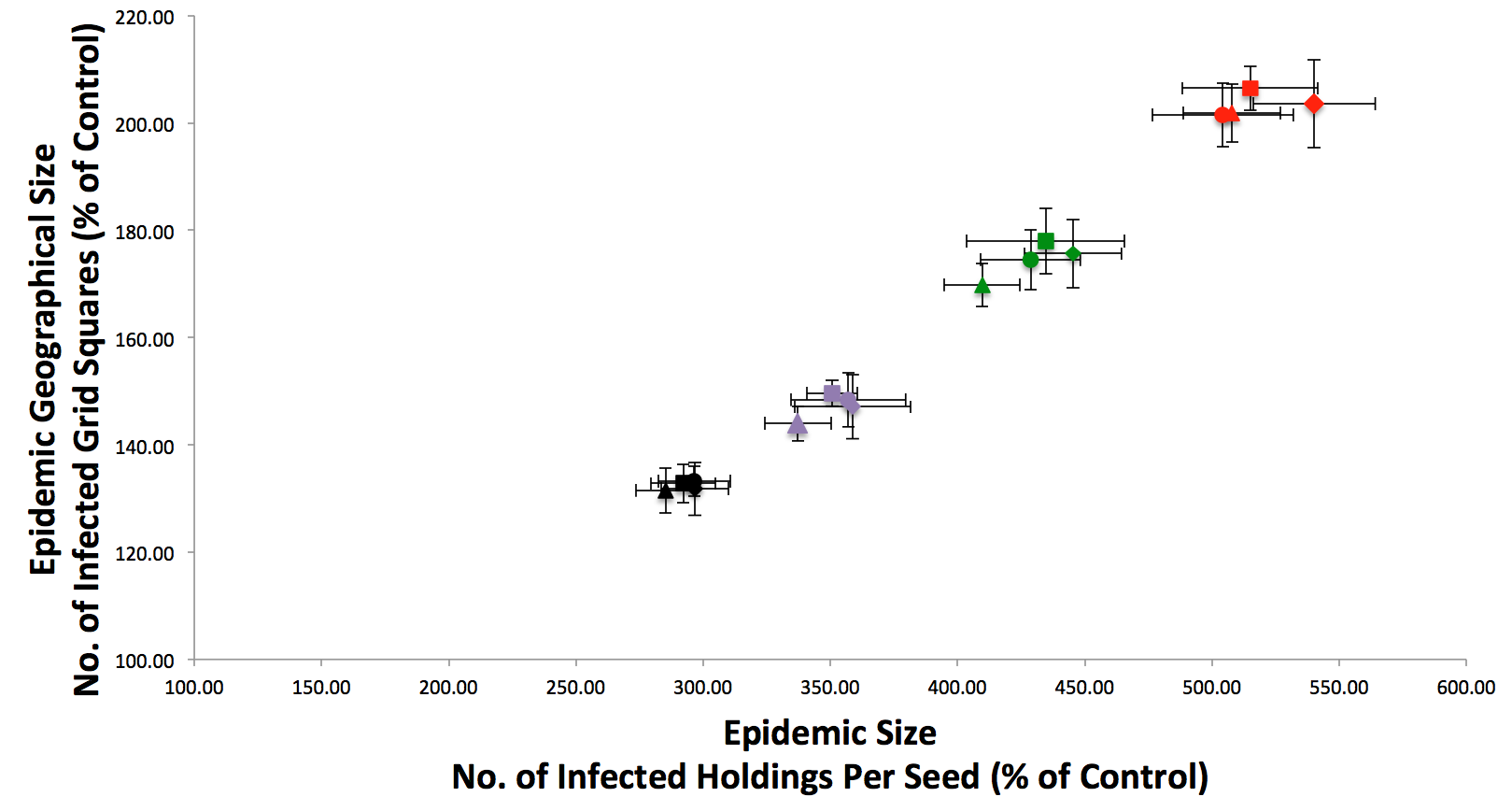

Supplement: Figure S5 — Comparison of epidemic sizes and geographical spread with SOAs and CTS Chains combined. SOAs and CTS Chains were combined to form one large set of SOA/Chain linked holdings. The model was run including the combined SOAs/Chains, with the following distance limits on intra-Chain spread: No Limit (red), 50 km (green), 16 km (purple), and 8 km (black). Epidemic size (number of infected holdings per seed) and geographical size (number of infected grid squares per seed) were firstly transformed into percentages of the control epidemic size from the same time of year. Secondly, averages were then obtained for the four quarters of the year (Jan-Feb-Mar [squares], Apr-May-Jun [circles], Jul-Aug-Sep [triangles], Oct-Nov-Dec [diamonds]) giving four data points for each scenario. Error bars are associated with each quarterly average value that represent the 95% confidence intervals for that quarter, assuming a normal distribution and a standard deviation calculated from that quarter's data. (TIFF) [file pone.0035089.s006.tiff]
